# Spermidine treatment: induction of autophagy but also apoptosis?

**DOI:** 10.1186/s13041-024-01085-7

**Published:** 2024-03-05

**Authors:** Maxinne Watchon, Amanda L. Wright, Holly I. Ahel, Katherine J. Robinson, Stuart K. Plenderleith, Andrea Kuriakose, Kristy C. Yuan, Angela S. Laird

**Affiliations:** Motor Neuron Disease Research Centre, Macquarie Medical School, Faculty of Medicine, Health and Human Sciences, Macquarie University, Level 1, 75 Talavera Rd, 2109 Sydney, NSW Australia

**Keywords:** Machado-Joseph disease, Neurodegeneration, PolyQ, Spinocerebellar ataxia type 3, Autophagy, Zebrafish, Trinucleotide repeat disease

## Abstract

Machado-Joseph disease (MJD), also known as spinocerebellar ataxia type 3, is a fatal neurodegenerative disease that causes loss of balance and motor co-ordination, eventually leading to paralysis. It is caused by the autosomal dominant inheritance of a long CAG trinucleotide repeat sequence within the ATXN3 gene, encoding for an expanded polyglutamine (polyQ) repeat sequence within the ataxin-3 protein. Ataxin-3 containing an expanded polyQ repeat is known to be highly prone to intraneuronal aggregation, and previous studies have demonstrated that protein quality control pathways, such as autophagy, are impaired in MJD patients and animal models of the disease. In this study, we tested the therapeutic potential of spermidine on zebrafish and rodent models of MJD to determine its capacity to induce autophagy and improve functional output. Spermidine treatment of transgenic MJD zebrafish induced autophagy and resulted in increased distances swum by the MJD zebrafish. Interestingly, treatment of the CMVMJD135 mouse model of MJD with spermidine added to drinking water did not produce any improvement in motor behaviour assays, neurological testing or neuropathology. In fact, wild type mice treated with spermidine were found to have decreased rotarod performance when compared to control animals. Immunoblot analysis of protein lysates extracted from mouse cerebellar tissue found little differences between the groups, except for an increased level of phospho-ULK1 in spermidine treated animals, suggesting that autophagy was indeed induced. As we detected decreased motor performance in wild type mice following treatment with spermidine, we conducted follow up studies into the effects of spermidine treatment in zebrafish. Interestingly, we found that in addition to inducing autophagy, spermidine treatment also induced apoptosis, particularly in wild type zebrafish. These findings suggest that spermidine treatment may not be therapeutically beneficial for the treatment of MJD, and in fact warrants caution due to the potential negative side effects caused by induction of apoptosis.

**Supplementary Information:**

The online version contains supplementary material available at 10.1186/s13041-024-01085-7.

## Introduction

Machado-Joseph disease (MJD), also known as spinocerebellar ataxia type 3, is a fatal neurodegenerative disease that affects the control and coordination of muscles. It is caused by the autosomal dominant inheritance of the *ATXN3* gene containing a CAG repeat trinucleotide sequence that encodes for the polyglutamine (polyQ) sequence in the ataxin-3 protein. In healthy individuals, the polyQ repeat length within the ataxin-3 protein ranges from 12 to 40 repeats, whilst MJD patients are found to inherit more than 40 polyQ repeats [1]. Long polyQ peptides are known to be highly aggregation prone [2] and diseases caused by polyQ repeat expansion are usually characterised by the presence of polyQ-containing protein aggregates. In MJD, ataxin-3 positive protein aggregates are often found to be mislocalised to the nucleus of neurons, located within neuronal intranuclear inclusions [3].

The autophagy pathway is a cellular protein quality control pathway that aids in degradation of aggregated proteins. This process involves the envelopment of cytoplasmic debris within a structure called a phagophore, that matures into a double membranous structure known as the autophagosome. Finally fusion of the autophagosome with a lysosome, containing hydrolytic enzymes, degrades the contents within leading to removal of unwanted material [4]. Previous studies have revealed that autophagic activity is likely to be impaired in MJD. For example, immunohistochemical staining of MJD patient brain tissue has revealed accumulation of autophagy substrates including p62, LC3 and Atg16 [5–7]. Furthermore, MJD patient fibroblasts exhibit reduced beclin-1 levels [7] and impaired autophagic dynamics, failing to produce mature autophagosomes [8]. Our team recently identified impaired autophagy within cellular and zebrafish models of MJD [9]. These findings of defective autophagic machinery are not unique to MJD and have also been proposed to occur in other neurodegenerative diseases such as Parkinson’s disease [10], amyotrophic lateral sclerosis [11] and Alzheimer’s disease [12].

In this study, we investigated whether treatment with spermidine, a compound known to induce activity of the autophagy pathway, would have beneficial effects on in vivo models of MJD. Spermidine is a polyamine found naturally in food and within the body and is thought to play a critical role in a myriad of cellular functions including cell proliferation, differentiation and prevention of cell senescence [13, 14]. MJD symptoms typically develop once patients reach adulthood, which could coincide with the natural decline of spermidine and thus potential decreases in autophagic activity. Spermidine administration has been demonstrated to induce autophagy and cell survival in several cell culture and animal model studies [15]. We hypothesise that early supplementation of spermidine may enhance/sustain autophagic activity and thus may provide therapeutic benefit by preventing the formation and accumulation of ataxin-3 protein aggregates.

Here, we found that spermidine was able to induce autophagy, rescue the motor impairment and reduce levels of soluble human ataxin-3 protein in the MJD zebrafish. When investigating spermidine treatment further within a murine MJD model, we found that treatment did not improve the motor activity of the MJD mice, nor reduce the presence of ataxin-3 protein species within the brain.

## Methods

### Husbandry, generation and maintenance of transgenic zebrafish expressing ATXN3

Experiments carried out using animals in this study were performed in accordance with the animal ethics committee of Macquarie University (ARA: 2016/004 and 2017/019). Zebrafish were housed in a standard recirculating aquarium system at 28.5 ^o^C with a 14:10 light:dark cycle and fed daily with artemia and standard pellet. Transgenic MJD zebrafish used in this study had been used and described previously [16]. This transgenic line resulted from crossing driver line zebrafish expressing Tg(*elavl3*:Gal4-VP16; mCherry) with responder line zebrafish, Tg(UAS:dsRED,EGFP-ATXN3_Q23) or Tg(UAS:dsRED,EGFP-ATXN3_Q84), to generate zebrafish carrying neuronal expression of human ataxin-3 containing either 23Q or 84Q fused to EGFP. Drug treatment studies utilised the resulting offspring (F2) of in-crossing the F1 generation zebrafish.

In experiments that required human ataxin-3 expression without an EGFP-fusion protein, we used a transgenic zebrafish line ubiquitously expressing human ataxin-3 with C-terminal BFP fusion protein. The expression construct was generated using ubiquitin protomer sequence and BFP fusion protein sequence (both from the Zebrafish Tol2 Gateway-Compatible kit (gift from Emily Don, Addgene kit #1,000,000,087, [17]) together with human ATXN3 sequence containing 23 or 84 CAG repeats (pcDNA3-myc-Ataxin3Q84 was a gift from Henry Paulson, Addgene plasmid # 22,124; http://n2t.net/addgene:22124; RRID:Addgene_22124 [18]). These constructs were injected into one-cell stage embryos together with transposase mRNA and embryos carrying BFP (blue) expression were raised to adulthood. Potential founders were outcrossed, and their offspring screened for ubiquitous BFP expression. Tg(-3.5ubb:has.ATXN3-23Q-mTagBFP) and Tg(-3.5ubb:has.ATXN3-84Q-mTagBFP). Offspring from F3 generation of these transgenic lines (named Tg(-3.5ubb:has.ATXN3-23Q-mTagBFP) and Tg(-3u.5ubb:has.ATXN3-84Q-mTagBFP)) were used for the acridine orange staining experiment.

### Drug treatment of zebrafish models of MJD

Zebrafish embryos positive for the ATXN3-84Q transgene were identified via the expression of fluorophores (EGFP and dsRED), at 24 h post fertilisation (hpf). Positive embryos were treated with a single administration of spermidine (62.5 µM, 125 µM and 250 µM, solubilised in E3 medium), or chloroquine (1.5 mM and 3 mM, solubilised in E3 medium) and a vehicle control (E3 medium only). Spermidine was purchased from Cayman Chemicals, whilst chloroquine was purchased from Sigma Aldrich. Control (vehicle) treated animals (all genotypes: non-transgenic siblings, EGFP-Ataxin-3 23Q and 84Q) received the equivalent volume of appropriate vehicle (E3 medium). Zebrafish larvae were exposed to the drug compound until 6 days post fertilisation (dpf), at which point motor behaviour analysis and generation of protein lysates for western blotting were performed. Morphologically abnormal larvae were not included in the study. Approximately 20–25 embryos were treated per group per experiment.

### Motor behavioural assay of zebrafish larvae

Zebrafish behavioural analysis was performed in the Zebrabox using the Viewpoint Zebralab tracking software. Tracking of 6 dpf larvae was conducted by placement into rows of a 24-well plate, with experimental groups allocated to rows in a randomised manner to eliminate location bias. The multi-well plate was then acclimatised in the Zebrabox for 20 min. Larvae were then exposed to conditions of 6 min light and 4 min darkness. The total distance travelled in periods of darkness were calculated and analysed.

### Acridine orange staining of the Ubb ATXN3 BFP zebrafish

Transgenic zebrafish ubiquitously expressing human ataxin-3 (Ubb-ATXN3 23Q BFP and Ubb ATXN3 84Q BFP) as well as wild-type (WTTAB) controls were used for this experiment. Transgenic zebrafish embryos were confirmed positive for the human ATXN3 transgene with the identification of the BFP fluorophore at 24 hpf. Positive embryos, as well as wild-type embryo controls, were treated either with spermidine (250 µM) or vehicle (E3 medium) control from 24 hpf. Zebrafish aged 48 hpf were then treated with acridine orange (diluted into E3 medium; 5 µg/mL) for 10 min before washed in E3 ten times for two minutes each. Zebrafish larvae were individually placed into a 96-multi black well (clear bottom) plate and anesthetised with tricaine. Zebrafish larvae were imaged for GFP using a Leica DMi8 inverted microscope (at 5x magnification) equipped with Leica DFC 365 FX camera (Leica Microsystems). Apoptosis was then measured via GFP fluorescence intensity using Image J [19]. Approximately 15–20 embryos were used per replicate.

### CMVMJD135 mouse treatment study

Within this study, we utilised male CMVMJD135 transgenic mice that ubiquitously express human mutant (expanded) *ATXN3* driven by a CMV promotor at near endogenous levels [20]. The procedures performed within this study were approved by the Macquarie University Animal Ethics Committee (ARA 2017/044). Tail tissue samples were taken at approximately 2–3 weeks of age and DNA was extracted to confirm presence or absence of the CMV promoter. DNA from animals found to be CMV + then underwent further analysis to sequence the CAG repeat region of the human ATXN3 gene. CMVMJD135 mice with a repeat length of 134 to 141 CAGs were recruited into this study.

The CMVMJD135 transgenic colony was maintained at Australian BioResources (Moss Vale, Australia) and animals were shipped to Macquarie University at 4–5 weeks of age and commenced experimentation at 5.5 weeks of age. Mice were housed with 2–5 mice per cage throughout the duration of the study. A total of 64 male MJD and wildtype littermate mice were recruited into the study, with littermates randomly allocated to either spermidine or water treatment groups by experimenters not involved in the behavioural testing within the study. A total of 36 CMVMJD135 mice (*n* = 17, spermidine treated and *n* = 19 water treated) and 27 non-transgenic (*n* = 12 spermidine treated and *n* = 15 water treated) littermate controls were included in the study.

### Treatment administration of CMVMJD135 mice

Spermidine (Cayman Chemical) was dissolved in acidified drinking water at a concentration of 3 mM for mice whilst control mice received standard acidified drinking water. This dose and administration route was selected based on previous literature demonstrating induction of autophagy in mice following such administration [15, 21]. Mice had ad libitum access to drinking water which was refreshed three times per week (Monday, Wednesday, and Friday). The amount of water consumed by each cage of animals was recorded and compared to identify any difference between the mice administered spermidine versus standard water.

### Monitoring of neurological impairment and motor behaviour dysfunction

All mice underwent weekly monitoring for neurological impairment such ashind limb reflex, tremor and ataxic gait through use of a four-point scale (4 indicating highest impairment and 0 indicating no impairment), by a researcher blinded to experimental group. The total score of examined components was calculated for each animal, with twelve representing the highest score possible.

Each mouse was tested on a range of motor tasks including tests for balance and coordination assessment (accelerating rotarod and balance beam). Accelerating rotarod testing measured the latency to fall from an accelerating rotarod (Model 7650, Ugo Basile) with a starting speed of 4 rpm, with gradual acceleration to 40 rpm over the span of 300 s, with a time of 300 recorded for those that did not fall [22]. The beam test measured the latency to cross a square balance beam of 10 mm diameter, performed as described previously [20]. Rotarod and balance beam tests were each performed fortnightly, by a researcher blinded to experimental group, with three repeat tests per test day (each separated by five minutes of rest).

### Euthanasia and sample collection

Animals within the treatment study were divided into two cohorts, one cohort was euthanised at 18 weeks old, an age where disease onset is well established and the other at 25 weeks old, an age representing mid-stage disease. Animals were euthanised via sodium pentabarbitone overdose (300 mg/kg, IP) and underwent intracardiac perfusion with 0.9% saline, after which brain and spinal cord tissue was extracted. Brains were hemisected, with one hemisphere snap frozen in liquid nitrogen for protein extraction and the other hemisphere post-fixed in 4% paraformaldehyde for immunohistochemical processing.

### Immunohistochemical staining

Following 24 hours in 4% paraformaldehyde, brains were briefly washed in PBS (3 × 2 mins) then placed in 30% sucrose solution for a further 24 hours. Brains were then stored in PBS with 0.02% sodium azide for long-term storage before being embedded in Tissue Tek OCT compound (Sakura Finetek) and cryosectioned at 40 µm before storage in PBS with 0.02% sodium azide at 4°C until use. Collected cryosections underwent free floating DAB (3, 3’ diaminobenzidine) immunohistochemistry for ataxin-3 immunoreactivity. Cryosections were first incubated in 50% ethanol for 20 min at RT, then quenched in 3% H_2_O_2_ + 50% ethanol for 30 min at 4 °C prior to blocking in 3% bovine serum albumin (BSA) + 0.25% Triton X-100 in PBS for 1 h at RT. Sections were incubated for 24 h at 4 °C in rabbit anti-MJD (Ataxin-3 1:20,000, gift from Henry Paulson) diluted in blocking solution, followed by overnight incubation at 4 °C in biotinylated secondary antibody (1:2000 Vector Laboratories; goat anti-rabbit BA-1000) diluted in blocking solution. Sections were then incubated for 1 h at RT in Avidin-Biotin complex (Vector Laboratories, Vectastain Elite ABC Kit, Peroxidase Cat#PK-6100), prepared as per manufacturer instructions and left to complex for 30 min before application. Ataxin-3 immunolabelling was detected by incubating in DAB chromogen (Vector Laboratories, DAB Substrate Kit, Peroxidase Cat#SK-4100) for 10 min. Finally, sections were mounted onto glass slides and coverslipped with DAKO mounting media for imaging.

### Ataxin-3 imaging and manual quantification of aggregates

Imaging of ataxin-3 DAB sections was performed under brightfield (Zeiss Axio Imager Z3 microscope, 20x objective lens running Zen 3.5 software) at optimised exposure times for 18- and 25-week-old cohorts. Whole section images were captured at 20x magnification using the tiling function, and any stitching errors corrected post-acquisition using Zen lite software (Zeiss, Gottingen, Germany).

Manual blinded quantification of ataxin-3 aggregates within the medulla and pons was performed by a blinded investigator using the Multi-Point counting tool on ImageJ [23]. Required regions of interest were identified using the Allen Brain Atlas [24]. Three anatomically similar sections were chosen per animal for blinded manual aggregate counting within the medulla oblongata and the deep cerebellar nuclei (DCN). The sum of the aggregates from the three sections was taken as the total number of aggregates for the animal. Similarly, due to inconsistent number of hindbrain sections available, aggregates were counted within all anatomically similar sections containing the pons, and the average was taken instead of total aggregate count.

### Protein extraction and western blotting

Zebrafish larvae aged 6 dpf were prepared for protein extraction following euthanasia. Zebrafish larvae were placed into RIPA buffer containing protease and phosphatase inhibitors (Complete ULTRA tablets and PhosphoSTOP tablets, respectively; Roche), then homogenised manually using a dounce homogeniser.

Hemisected mouse cerebellar tissue was dissected and homogenised in 5 µL of RIPA buffer containing protease and phosphatase inhibitors per mg of wet tissue weight. Cerebellar tissue was then sonicated using an Omniruptor 250 Ultrasonic Homogeniser (Omni International).

Protein lysates from either zebrafish or mouse brain were centrifuged at 21,300 g at 4 °C for 20 min and clear supernatant collected. Total protein concentration of supernatants was determined using a Pierce BCA Protein Assay Kit (Thermo Fisher Scientific) and equal amounts of protein was prepared in Laemmli buffer (Bio-Rad) with NuPAGE Reducing Agent (Life Technologies). Denatured proteins were separated on NuPage™ 4–12% Bis-Tris gel (Thermo Fisher Scientific) or 4–15% (Biorad) gel through SDS-PAGE, then transferred onto a 0.45 μm PVDF membrane for immunoblot probing.

Antibodies used included rabbit anti-MJD (kind gift from H. Paulson), rabbit anti-phosphorylated ULK1 Ser777 (Merck Millipore), rabbit anti-ULK1 (Cell Signalling Technology), rabbit anti-beclin-1 (Proteintech), rabbit anti-p62 (MBL), mouse anti-p62 (Abcam), rabbit anti-LC3B (Abcam), rabbit anti-PARP (Cell Signalling Technology), rabbit anti-cleaved caspase 3 (Abcam), rabbit anti-caspase 3 (RnD) and mouse anti-GAPDH (Proteintech). Immunoblots were then probed with the appropriate secondary (Promega) and visualised by chemiluminescence (SuperSignal West Femto Maximum Sensitivity Substrate, Thermo Fisher) using either the ImageQuant LAS4000 or ImageQuant 800 Amersham. Band intensity was quantified using Image Studio Lite and the target protein was normalised against the loading control protein (GAPDH).

### Statistical analysis

Data analysis was performed using GraphPad Prism (version 9). Group comparisons within the zebrafish studies were analysed using a one-way ANOVA, followed by a Tukey post-hoc analysis. Densitometric analysis of autophagy proteins in the presence of an autophagy inducer versus the vehicle control were compared using a student t-test. Co-treatment studies involving spermidine and chloroquine were analysed using a two-way ANOVA followed by a Tukey post-hoc analysis. The behavioural data within the rodent study was analysed using two-way repeated measure ANOVA with experimental group and age as the factors, followed by Tukey post-hoc comparisons. Comparison of autophagy or apoptosis related protein expression in the mouse study used two-way ANOVA with genotypes and treatment as the effects and Tukey post-hoc comparison. Statistically significant differences are defined as *p* < 0.05.

## Results

### Treatment with spermidine alleviates motor phenotypes in the transgenic MJD zebrafish

**Fig. 1:**
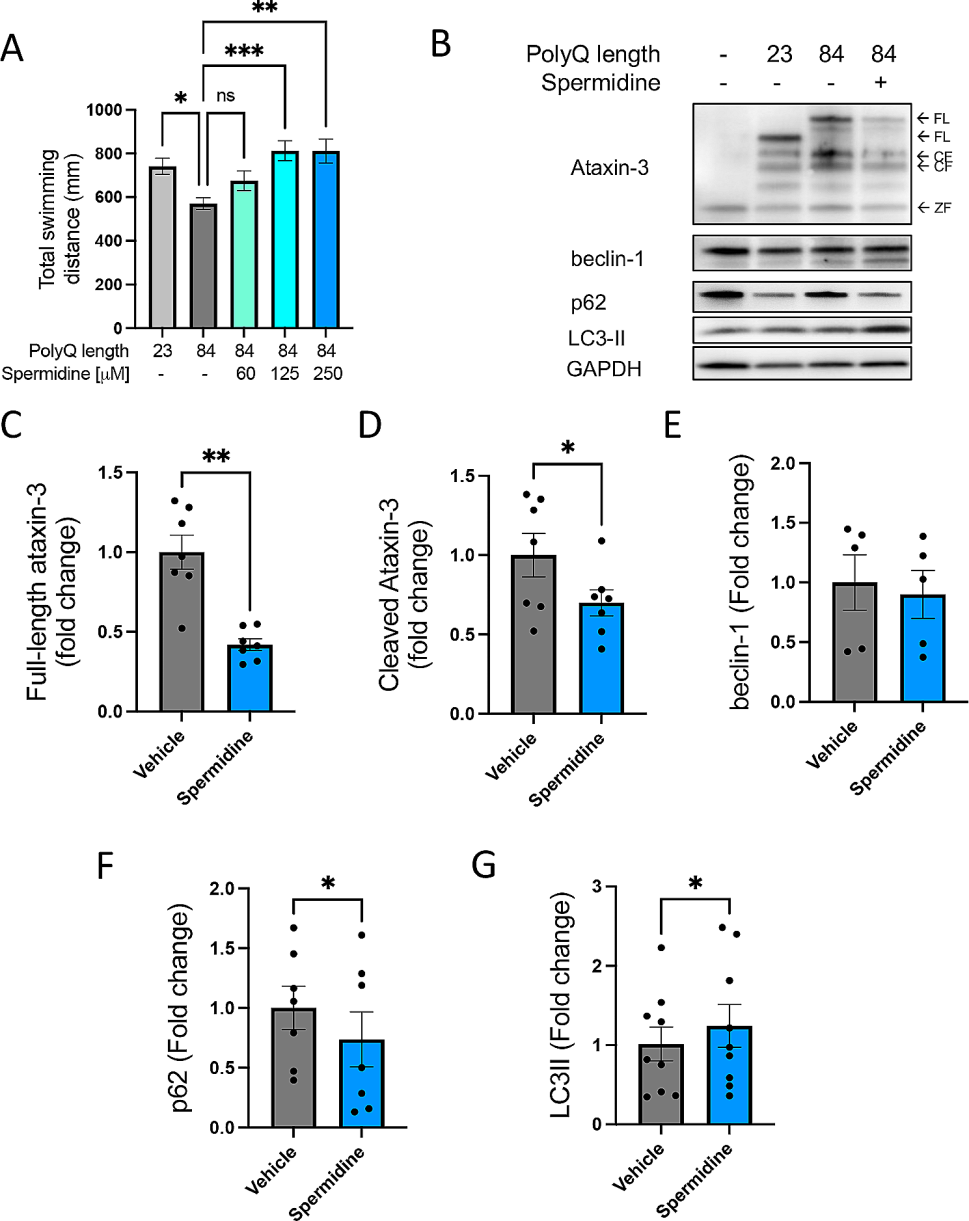
Spermidine treatment alleviates motor dysfunction in transgenic MJD zebrafish together with signs of autophagy induction. (**A**) Mutant ataxin-3 zebrafish (84Q) were treated with spermidine (60–250 µM) for 5 days and motor behaviour was measured at 6 dpf. Vehicle treated mutant ataxin-3 larvae swam shorter distances compared to the non-transgenic control and spermidine treatment rescued this phenotype (***p < 0.001 and *p < 0.05, *n* = 96–140). (**B**) Spermidine treated mutant ataxin-3 larvae were subjected to immunoblotting (**B**). Densitometric analysis of (**C**) full-length and (**D**) cleaved- human ataxin-3 revealed a significant decrease in ataxin-3 expression in spermidine treated EGFP-ATXN3 84Q fish compared to the vehicle treated mutants (** *p* = 0.002 and *p* = 0.020 respectively, *n* = 8–10). Various substrates of the autophagy pathway showed indications of positive autophagic flux. (**E**) No difference was found in levels of beclin-1, (**F**) p62 was found to be significantly decreased (*p = 0.0432) and (**G**) LC3II was found to be increased. (*p* = 0.0426, all *n* = 8–10). A one-way ANOVA followed by Tukey post hoc analysis and a paired student t-test was utilised for statistical analysis. Data represents mean ± SEM. Data points reflected in C-G are experimental treatments of approximately 20–25 larvae

Zebrafish larvae neuronally expressing EGFP-Ataxin-3 84Q swam shorter distances during motor testing at 6 dpf than those expressing EGFP-Ataxin-3 23Q (wild-type) or non-transgenic controls [16]. Here, we treated the MJD zebrafish (EGFP-Ataxin-3 84Q) with spermidine (62.5, 125 and 250 µM) from 24 h post fertilisation (hpf) to 6 days post fertilisation (dpf) to determine whether spermidine treatment could improvement motor ability. Treatment with 125 µM and 250 µM spermidine produced an increase in the distance swum by the ataxin-3 84Q zebrafish, compared to the vehicle treated mutant fish (Fig. 1A; *p* = 0.0005 and *p* = 0.001 respectively, *n* = 37–45). When these larvae were processed for immunoblotting, Ataxin-3 84Q zebrafish treated with 250 µM spermidine exhibited a robust decrease in the amount of full length and cleaved forms of human ataxin-3, compared to that in the vehicle treated mutant fish (Fig. 1B). Densitometric analysis of these immunoblots confirmed a significant reduction in full-length and cleaved ataxin-3 following spermidine treatment when compared to the vehicle control (Fig. 1C, D; *p* = 0.002 and *p* = 0.02 respectively). There is evidence that spermidine activates the autophagy pathway in models of neurodegenerative disease [25] therefore, markers of autophagy were examined. We have previously reported that the abundance of the autophagy substrate p62 is altered in the EGFP-ataxin-3 23Q zebrafish larvae and EGFP-ataxin-3 84Q adult zebrafish brains [9]. Here we found whilst levels of beclin-1 levels were not altered, the amount of p62 was reduced (*p* = 0.0432) and LC3II levels increased (*p* = 0.0426) in spermidine treated compared to vehicle treated mutant ataxin-3 fish (Fig. 1E-G). These results suggest that the spermidine treatment was likely inducing positive autophagic flux, causing mutant ataxin-3 levels to decrease.

### Beneficial effect of spermidine in MJD zebrafish is autophagy dependent

**Fig. 2:**
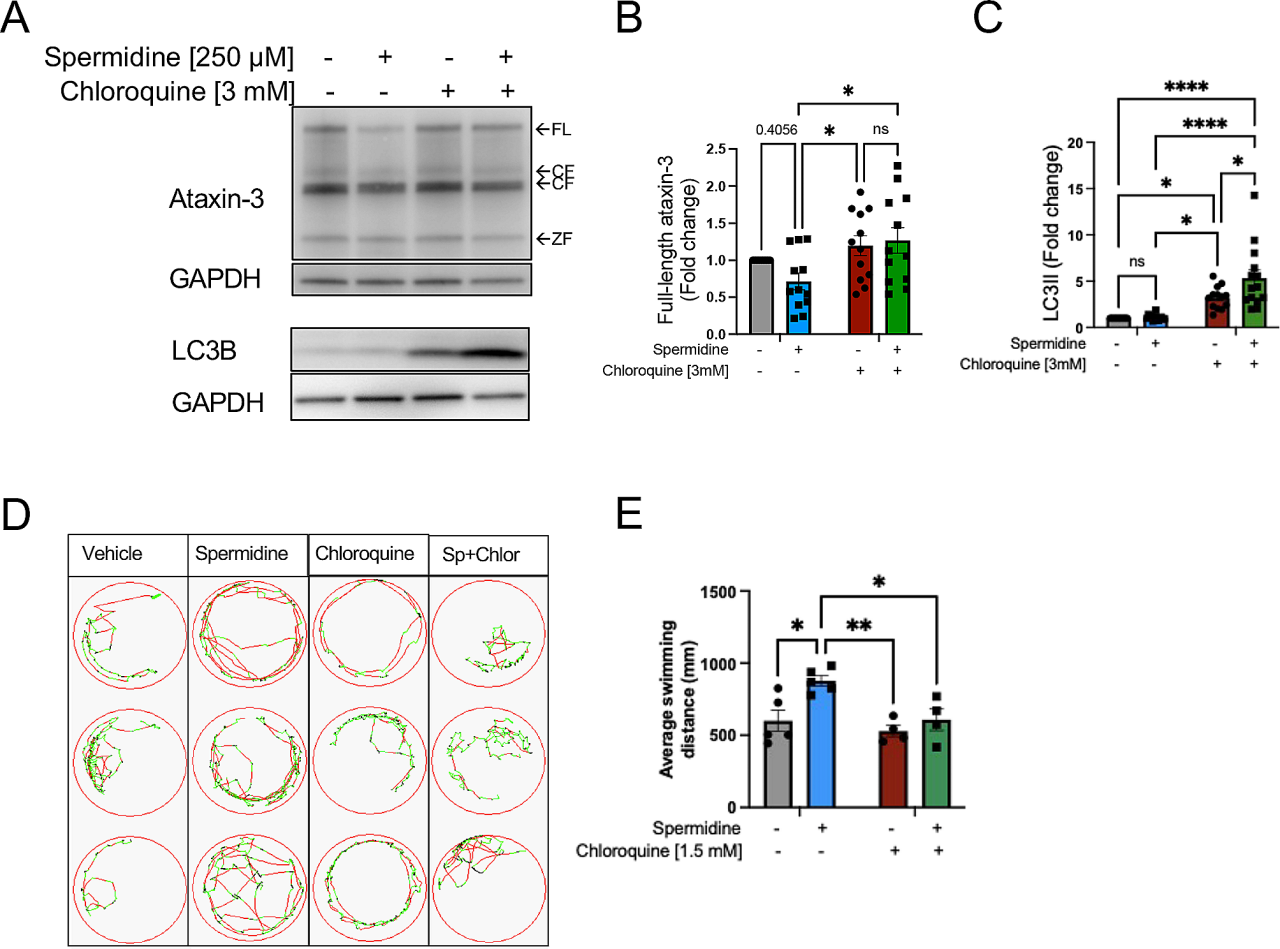
Spermidine treatment of MJD zebrafish resulted in autophagy induction and inhibition of the autophagy pathway prevented the improved swimming produced by spermidine treatment. (**A**) Cohorts of MJD zebrafish larvae were euthanised and processed for immunoblot analysis for ataxin-3, LC3B and GAPDH loading control. (**B**) Inhibition of the autophagy pathway with chloroquine resulted in a significant increase in full length ataxin-3 levels (*p* < 0.0459, *n* = 11–12). (**C**) Comparison of LC3II levels in lysates from zebrafish treated with chloroquine, versus those co-treated with chloroquine and spermidine revealed that spermidine treated produced a higher level of LC3II, indicative of induction of autophagy (*p* < 0.0167, *n* = 11–12). (**D**) Example swimming trajectories of MJD zebrafish larvae treated with vehicle, spermidine, chloroquine or spermidine + chloroquine (Sp + Chlor) during an escape response to darkness test showed that spermidine treated larvae spent more time swimming at fast speeds and less at slow speeds. Co-treatment with spermidine + chloroquine returned the swimming to more similar to the vehicle treated group. (**E**) Measurement of distances swum by the MJD zebrafish show that spermidine treatment significantly increased the distances swum but chloroquine co-treatment prevented the improvement produced by the spermidine treatment (*p* < 0.0295, *n* = 4–5). A two-way ANOVA was utilised for statistical analysis followed by Tukey post hoc. Data represents mean ± SEM. Data points reflected are experimental treatments of approximately 20–25 larvae

To confirm whether spermidine treatment had indeed been inducing autophagy, the MJD zebrafish were co-treated with spermidine (250 µM) and the autophagy inhibitor, chloroquine (1.5 mM and 3 mM). Motor behaviour and western blotting of ataxin-3 and autophagosome marker LC3II were then assessed. Whilst spermidine treatment alone appeared to decrease full-length ataxin-3 levels compared to the vehicle treated animals (Fig. 2A), densitometric analysis revealed it was not significant (Fig. 2B). However, chloroquine treatment alone (3 mM) as well as spermidine and chloroquine co-treatment had increased levels of full-length ataxin-3 in comparison to spermidine treatment alone (Fig. 2B; *p* = 0.0459 and *p* = 0.0165 respectively, *n* = 11–12). Chloroquine treatment (3 mM) alone produced autophagy inhibition, indicated by elevated levels of LC3II compared to the vehicle treated and spermidine treated EGFP-Ataxin-3 84Q fish (Fig. 2C; *p* = 0.016 and *p* = 0.020 respectively, *n* = 13–14). LC3II levels were further increased following co-treatment with spermidine and chloroquine compared to chloroquine treatment alone, spermidine treatment alone and vehicle treatment (Fig. 2C; *p* = 0.024, *p* < 0.0001 and *p* < 0.0001 respectively). The further increase in LC3II levels following spermidine and chloroquine co-treatment suggests the generation of autophagosomes triggered by spermidine treatment is also prevented by degradation through the chloroquine co-treatment, thus highlighting that spermidine is indeed inducing autophagy.

Next, we sought to investigate whether co-treatment with chloroquine and spermidine prevented the amelioration of motor function elicited by spermidine treatment. We had identified within the wild-type zebrafish that treatment with 3 mM chloroquine, required to block autophagy to measure autophagy flux, decreased the swimming of the zebrafish, whilst 1.5 mM chloroquine did not (Supplementary Fig. 1A). Therefore, a lower concentration of chloroquine (1.5 mM) was used to test whether the improved motor behaviour produced by spermidine treatment was autophagy dependent. As noted above, MJD zebrafish larvae treated with spermidine swam longer distances compared to those treated with vehicle and chloroquine treatment alone, and co-treatment with spermidine and chloroquine also significantly decreased the distances swum by the larvae (Fig. 2D, E; *p* < 0.0295, *n* = 4–5 experimental replicates). This result indicates that the rescue of the motor dysfunction in the mutant ataxin-3 zebrafish produced by spermidine treatment was dependent on autophagic activity.

### Spermidine treatment did not decrease motor impairment in MJD mice and in fact worsened it for wild type mice

**Fig. 3:**
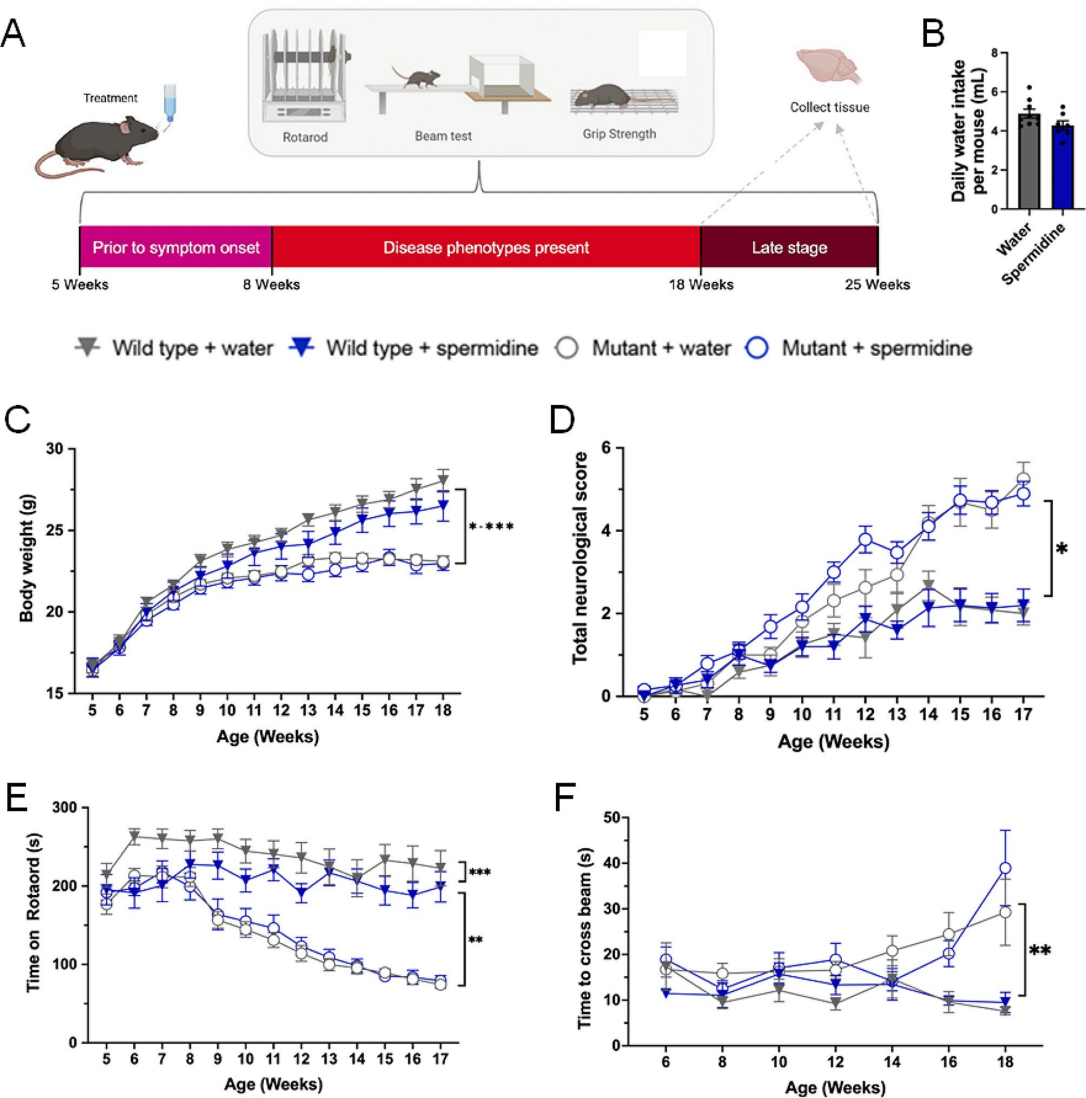
Spermidine treatment did not improve the motor impairment of CMVMJD135 mice developed motor impairment. (**A**) A schematic diagram illustrates that CMVMJD135 mice were treated with spermidine (3 mM) within their drinking water from 5 to 25 weeks old. Behavioural testing was conducted throughout the study and a subset of animals were euthanised at 18 weeks old and another at 25 weeks old. (**B**) Comparison of water intake between mice treated with spermidine and water found no significant difference (*p* = 0.809). (**C**) MJD mice had lower body weights than wild-type mice from 9 weeks old onwards (*p* < 0.0236), and although spermidine treatment appeared to cause decreased weights in the wild type mice, this was not a statistically significant effect. (**D**) Whilst mutant CMVMJD135 mice developed increased neurological scores, indicative of neurological impairment, from around 9 weeks old onwards (*p < 0.0193), treatment with spermidine did not improve this impairment (it worsened it at 7 and 12 weeks old (*p* < 0.0402). (**E**) MJD mice had decreased latencies on the rotarod compared to wild-type mice from around 9 weeks old (**p < 0.0001). Treatment with spermidine did not increase the latency before falling from an accelerating rotarod. In fact, treatment with spermidine treatment decreased the latency to fall in wild type mice compared to water treated wild type mice at 6 and 7 weeks old (***p < 0.0331). (**F**) MJD mice took longer to cross the balance beam than wild-type mice at 16 and 18 weeks old (**p < 0.0106). Spermidine treatment did not affect the time taken by mice to cross a balance beam, regardless of genotype. A repeated one-way ANOVA was utilised for statistical analysis followed by Tukey post hoc. Data represents mean ± SEM. Biorender.com was used to make the image in panel A

We next investigated whether spermidine was beneficial as a treatment within the CMVMJD135 murine model of MJD. Spermidine (equivalent to 3 mM) or vehicle treatment was continuously given to the CMVMJD135 mice and age matched wild-type controls via drinking water, from a pre-symptomatic age of 5 weeks until symptomatic stage (18 weeks) or mid- disease stage(25 weeks), at which points brain tissue was collected (Fig. 3A). Daily water intake per cage of mice was recorded and no difference was found between mice treated with water and spermidine (*p* = 0.809, Fig. 3B). Two-way ANOVA analysis of the body weight of the mice revealed an effect of experimental group (*p* < 0.0001) and age (*p* < 0.0001) with an interaction effect of age and group (*p* < 0.0001), on the weight of the animals (Fig. 3C). MJD mice had lower weights than wild-type mice from 9 weeks old onwards (*p* < 0.0236), and although spermidine treatment appeared to cause decreased weights in the wild type mice, this was not a statistically significant effect. Neurological scores assigned to each mouse (by a researcher blinded to experimental group) for their neurological function revealed an effect of experimental group (*p* < 0.0001) and age (*p* < 0.0001) with an interaction effect of age and group (*p* < 0.0001). MJD mice displayed elevated neurological scores compared to wild-type mice, indicative of commencement of MJD symptoms (abnormal hindlimb reflex, tremor or gait abnormalities) from around 9 weeks of age (Fig. 3D; *p* < 0.0193). Spermidine treatment failed to improve neurological function of the CMVMJD135 mice (compared to water treatment), with post-hoc analysis indicating that the spermidine treated MJD mice had poorer neurological scores than water treated MJD mice at 7 and 12 weeks old (*p* < 0.0402).

Mice were tested on the accelerating rotarod as a means of examining balance and coordination. The latency to fall from the rotarod was recorded and the average of three tests was calculated. Two-way repeated measure ANOVA analysis found a significant effect of experimental group (*p* < 0.0001) and age (*p* < 0.0001), with an interaction effect for age and group (Fig. 3E; *p* < 0.0001). Tukey’s post-hoc analysis showed that spermidine treated wild-type mice exhibited shorter rotarod latencies than water treated wild-type mice at weeks 6–7 (*p* < 0.0331) and water treated wild-type mice had longer latencies than water treated mutant mice from week 9 onwards (*p* < 0.0001). Within the balance beam test, two-way repeated measure ANOVA found an effect of experimental group (*p* < 0.0001) and an interaction effect for age and group (*p* = 0.0055). Tukey’s post-hoc analysis showed that the differences were present between the wild-type and MJD mice at weeks 16 and 18 (Fig. 3F; *p* < 0.0106).

### Effect of spermidine treatment on MJD-related neuropathology

**Fig. 4:**
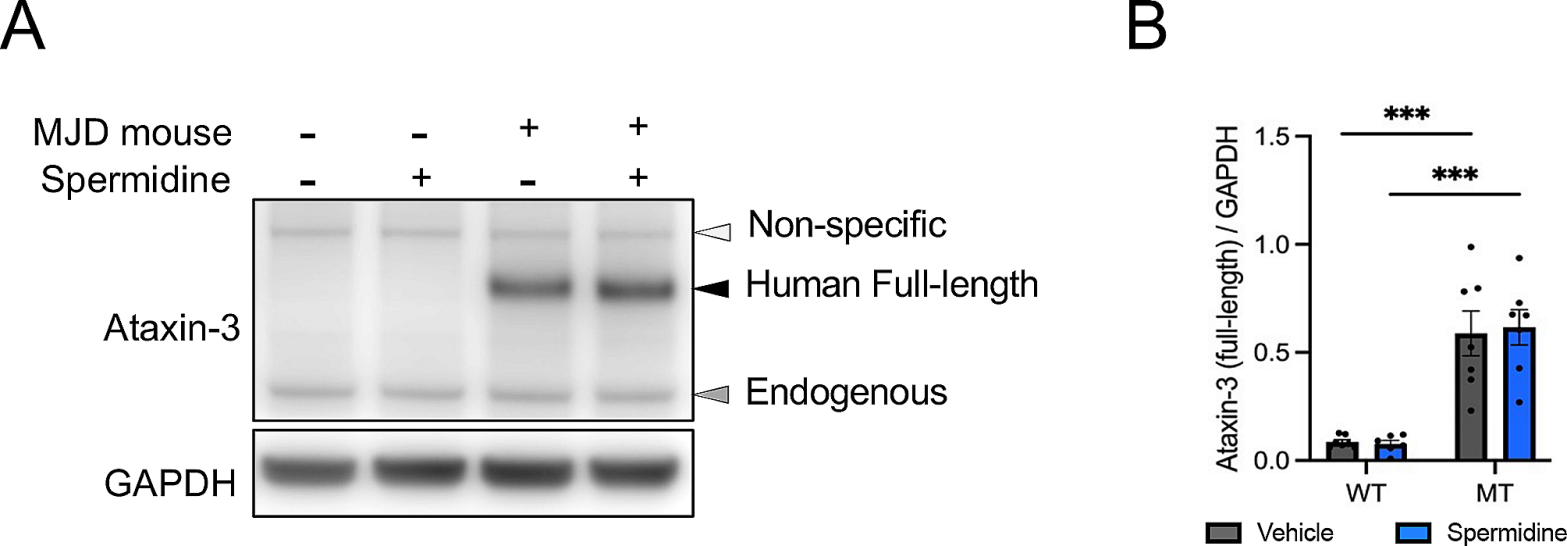
Immunoblotting of wild-type and transgenic CMVMJD135 mice confirms increased expression of full-length ataxin-3 in the cerebellum of 18-week-old MJD mice. (**A**) Representative western blot of 18-week-old wild-type and MJD mouse cerebellum following oral vehicle or spermidine supplementation confirming expression of full-length and endogenous ataxin-3. (**B**) Quantification of full-length ataxin-3 expression shows significant increase in the cerebellum of 18-week-old MJD mice compared to wild-type mice (*p* < 0.0001, *n* = 6–7). Expression of full-length ataxin-3 is not significantly altered following vehicle or spermidine treatment. A two-way ANOVA was utilised for statistical analysis followed by Tukey post hoc. Data represents means ± SEM. *** Represents *p* < 0.0001

Immunoblotting of protein lysates of the cerebellar tissue from MJD mice dissected at autopsy at 18 weeks revealed human ataxin-3 protein expression in the MJD mice as well as endogenous ataxin-3 in both the MJD mice and non-transgenic controls (Fig. 4A). Spermidine treatment, regardless of genotype, did not affect ataxin-3 protein levels (Fig. 4B).

**Fig. 5:**
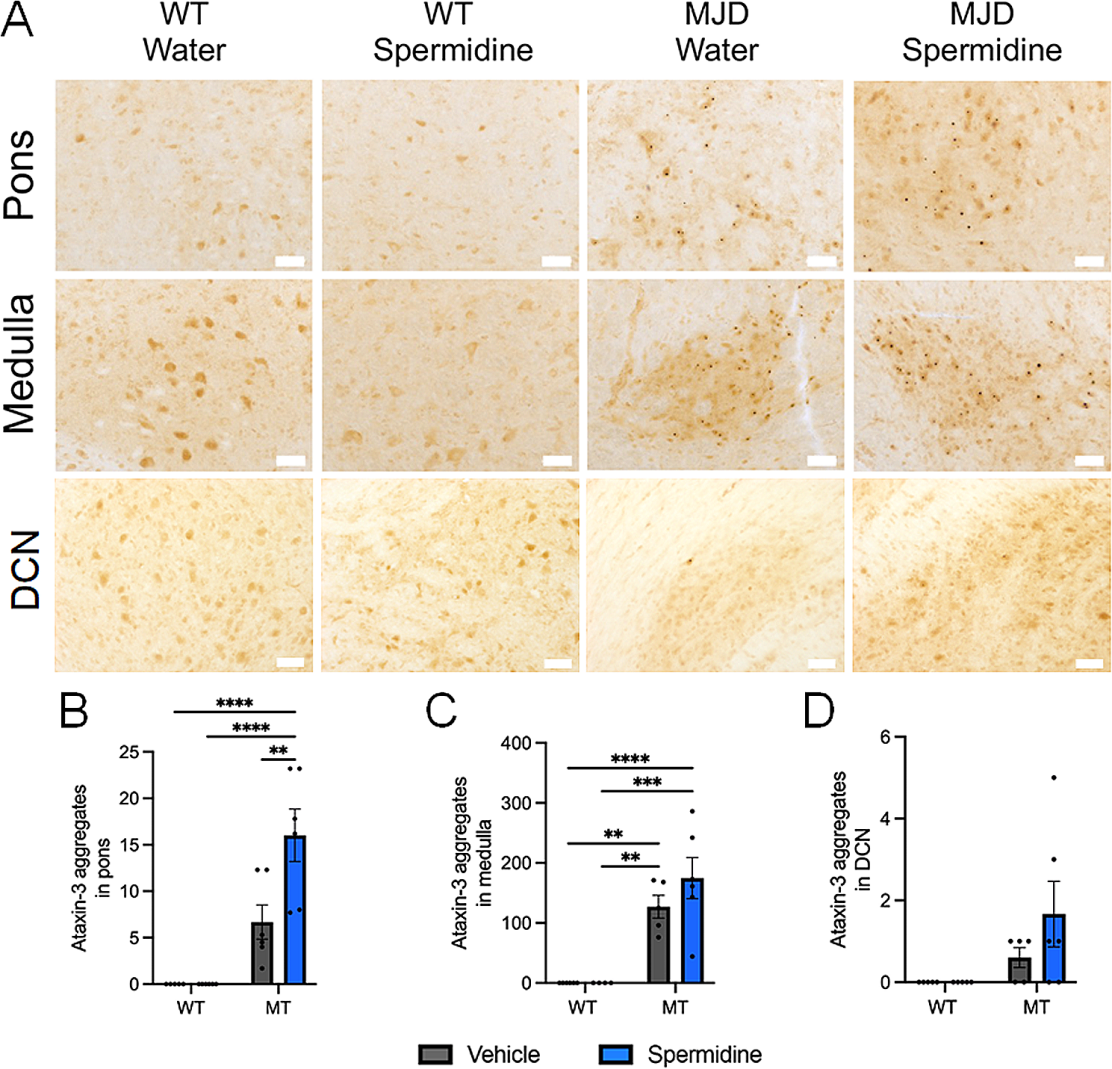
Spermidine treatment did not affect presence of protein aggregates within the pons and medulla of 18-week-old CMVMJD135 mice. (**A**) Immunohistochemical staining for ataxin-3 in sections from the pons and medulla of wild type (WT) and CMVMJD135 (MJD) mice revealed that the MJD mice harboured ataxin-3-positive protein aggregates. Scale bars indicate 50 μm. (**B**) MJD mice exhibited more ataxin-3 aggregates than WT mice within the pons (*p* < 0.0001) and spermidine treatment had no effect on the number of ataxin-3 aggregates present. (**C**) MJD mice exhibited more ataxin-3 aggregates within the medulla oblongata than WT mice (*p* < 0.0001) and spermidine treatment had no beneficial effect on the number of ataxin-3 aggregates present, in fact greater aggregate numbers were found in the spermidine treated MJD mice compared to those treated with water (*p* = 0.0059). (**D**) More aggregates were found within the DCN region of MJD mice than WT mice (*p* = 0.0308), but spermidine treatment did not affect the number of aggregates present. A two-way ANOVA was utilised for statistical analysis followed by Tukey post hoc. The p-values marked on the graphs represent the Tukey test results. Data represents mean ± SEM

Immunohistochemical staining for human ataxin-3 in the brain of 18-week-old mice revealed the presence of ataxin-3-positive aggregates within various regions of the MJD mouse brain, including the pons, medulla oblongata and deep cerebellar nuclei (DCN) (Fig. 5A). Blinded manual counting of the ataxin-3 aggregates within the pons and medulla oblongata revealed more ataxin-3-positive aggregates in the brains of MJD mice compared to WT controls, confirmed by the finding of a significant effect of genotype on the number of aggregates present (Fig. 5B, C; *p* = 0.0001 for each). Few aggregates were counted within the deep cerebellar nuclei of MJD mice, but no ataxin-3 aggregates were found within the DCN of WT mice, resulting in a significant effect of genotype (*p* = 0.0308) (Fig. 5D). Spermidine treatment had no effect on the presence of ataxin-3 aggregates within any of these regions, except in the pons where spermidine treated mice exhibited more aggregates than water treated MJD mice (*p* = 0.0059, Tukey test). Similar results were found following immunohistochemical staining of the brains of 25-week-old mice, where MJD animals had a greater number of aggregates in each region (*p* < 0.0482) and spermidine treatment had no effects on aggregate numbers (Supplementary Fig. 2A-D).

### Examination of autophagy and apoptotic pathway alterations after spermidine treatment in MJD mice

**Fig. 6:**
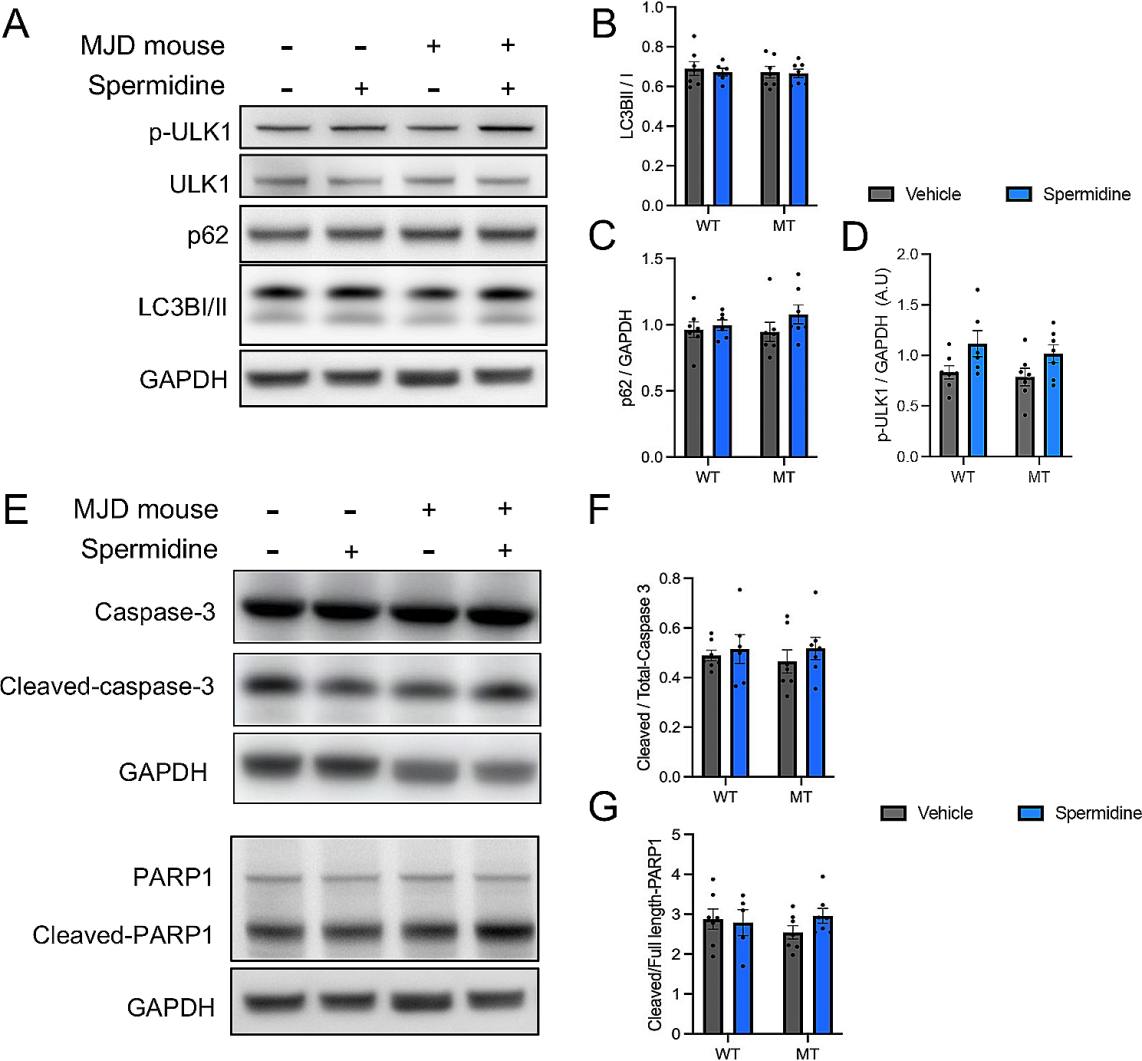
Spermidine treatment within drinking water promotes autophagy through increased phosphorylation of ULK1 in mice independent of genotype but does not induce apoptosis. (**A**) Representative western blot of cerebellum from wild-type and MJD mice treated with vehicle or spermidine and probed for markers of autophagy p-ULK1, ULK, p62 and LC3B. (**B**) Quantification of the ratio between LC3II/I showed no significant differences after spermidine treatment. (**C**) Quantification of p62 showed no significant differences following spermidine treatment (**D**) Simple main effects analysis of p-ULK1 levels demonstrated a significant increase following spermidine treatment ( *p* = 0.0109, *n* = 6–7), independent of genotype. (**E**) Representative western blot of wild-type and MJD mice treated with vehicle or spermidine and probed for markers of apoptosis including cleaved- and total-caspase 3 and downstream substrates cleaved- and total-PARP1. (**F**) Quantification of cleaved- and total-caspase 3 showed no significant differences following spermidine treatment. (**G**) Quantification of the ratio between cleaved- and full-length-PARP1 revealed no significant differences following spermidine treatment. A two-way ANOVA was utilised for statistical analysis followed by Tukey post hoc analysis. Data represents mean ± SEM. * Represents *p* < 0.05

As stated above, spermidine treatment in the MJD zebrafish was able to induce activity of the autophagy pathway. We also sought to investigate whether the autophagy pathway was also induced in the transgenic MJD mice. Therefore, we performed immunoblot analysis on protein lysates extracted from the cerebellum of the 18-week old mice at autopsy. When detecting markers of autophagy via immunoblotting, phosphorylated-ULK ser777 (pULK), ULK, p62 and LC3I/II, it did not appear to show differences between the groups, regardless of genotype or treatment (Fig. 6A). Analysis of LC3II/I levels or p62 confirmed there were no differences between the genotypes or treatment groups (Fig. 6B, D). On the other hand, pULK levels were increased in spermidine treated animals compared to its vehicle control, regardless of genotype (Fig. 6C; *p* = 0.0109).

To examine the level of apoptosis present following spermidine treatment, perhaps resulting from known links between autophagy activity and apoptosis, we performed immunoblot analysis on protein lysates extracted from the cerebellum of the mice at autopsy. Immunoblotting for apoptosis markers, cleaved caspase-3 and cleaved PARP1 revealed similarity in band intensity across the different treatments and genotypes (Fig. 6E). This was confirmed with densitometric analysis (Fig. 6F, G).

### Increased apoptosis following spermidine treatment in zebrafish

**Fig. 7:**
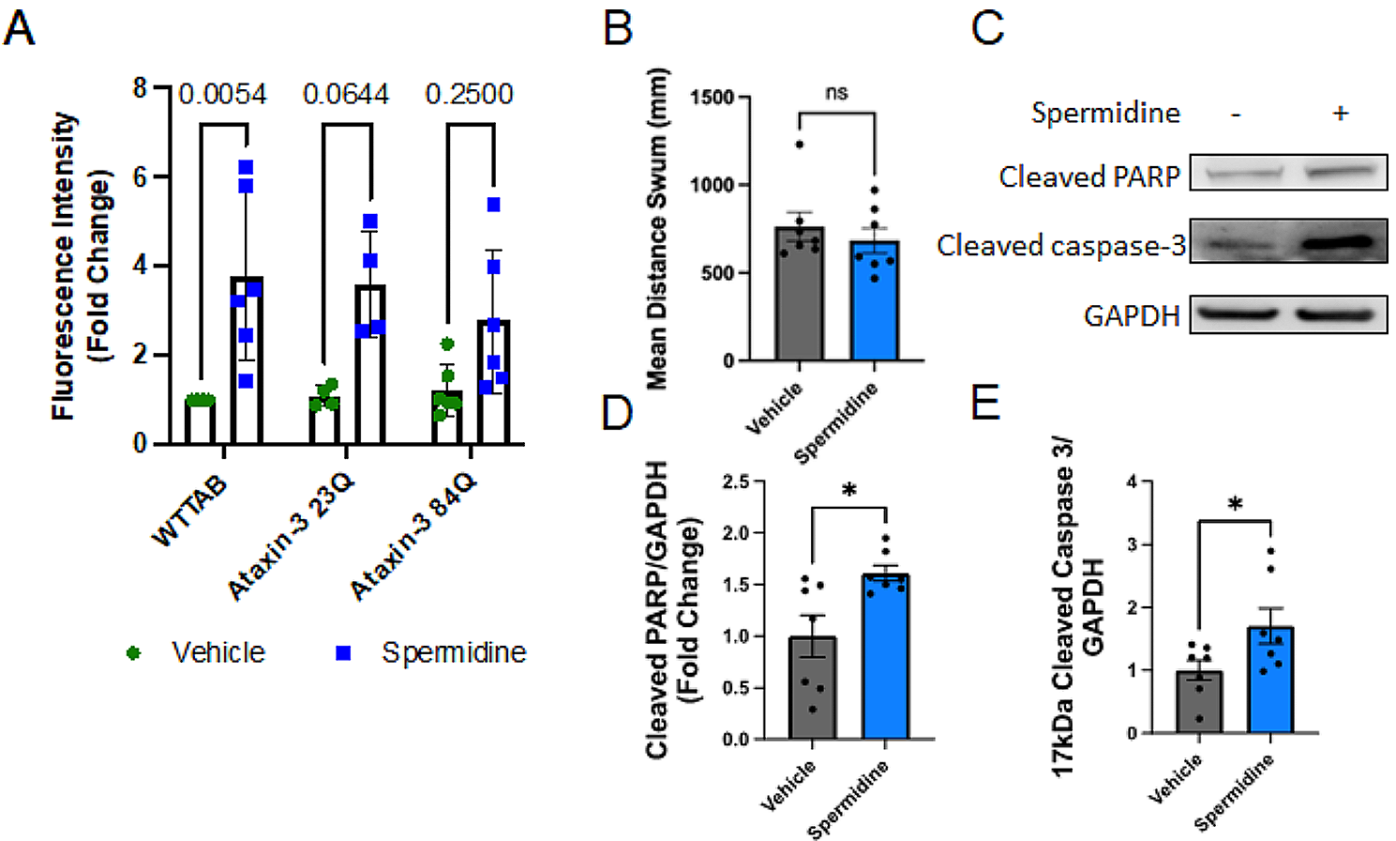
Signs of increased apoptosis in spermidine treated zebrafish larvae. (**A**) Non-transgenic (WTTAB) zebrafish larvae aged 2 days post fertlisation (dpf) showed increased acridine orange apoptosis staining following spermidine treatment (*p* = 0.0054). The increase in acridine orange staining in spermidine treated Ubb-Ataxin-3 84Q BFP zebrafish larvae was not statistically significant (*p* > 0.0644). (**B**) Comparison of the motor behaviour (total distance swum) of WT TAB zebrafish larvae treated with Vehicle or Spermidine found no difference between the groups. (**C**) Immunoblot analysis was performed to examine signs of apoptosis in non-transgenic zebrafish following spermidine treatment. (**D**) Spermidine treatment resulted in increased levels of cleaved PARP and (E) cleaved caspase 3, both signs of apoptosis (*p* = 0.0196 and *p* = 0.0255 respectively, *n* = 7). Statistical analysis performed were a two-way ANOVA followed by a Tukey post-hoc analysis and a paired Student t-test. Data represents mean ± SEM. Data points reflected are experimental treatments of approximately 20–25 larvae

Examination of the effect of spermidine treatment on zebrafish was examined via acridine orange staining and immunoblotting for the apoptosis markers, cleaved PARP and cleaved capsase 3. Spermidine treated wild-type fish from 1 to 2 dpf underwent acridine orange staining as a marker of apoptosis. This stain showed a significant effect of treatment (two-way ANOVA, *p* < 0.0001) with post-hoc analysis revealing significantly increased fluorescence intensity in spermidine treated wild-type zebrafish compared to vehicle treated wild-type fish (Fig. 7A; *p* = 0.0054). Interestingly, spermidine treatment failed to show increased GFP fluorescence intensity in the Ataxin-3 23Q or Ataxin-3 84Q zebrafish compared to their respective vehicle treated genotype.

To examine whether the increased apoptosis detected in the wild type zebrafish treated with spermidine had caused any motor impairment we compared the total distance swum by vehicle and spermidine treated wild type zebrafish larvae at 6 dpf. No difference was found between the two groups (*p* = 0.3829). To examine the positive acridine orange findings found in the 2 dpf zebrafish after spermidine treatment further, protein from 2 dpf wild-type zebrafish protein was extracted and immunoblotted for apoptosis markers, cleaved PARP and cleaved caspase-3. Spermidine treatment showed increased band intensity for cleaved PARP and cleaved caspase-3 (Fig. 7D). Densitometric analysis revealed a significant increase in cleaved PARP and cleaved caspase-3 (Fig. 7E, F; *p* = 0.0196 and *p* = 0.0255 respectively). Collectively, these results suggest that whilst spermidine treatment promotes induction of the autophagy pathway within the MJD zebrafish it produces increased apoptosis in non-transgenic zebrafish.

## Discussion

In this study, we tested the therapeutic potential of treating zebrafish and rodent models of MJD with spermidine, a naturally produced polyamine known to enhance autophagy activity [15]. Treatment of our transgenic MJD zebrafish with spermidine from 1 to 6 dpf resulted in improved motor behaviour at 6 dpf, as well as decreased human ataxin-3 levels on immunoblots. Immunoblot analysis for levels of autophagy substrates LC3II (unfortunately LC3I cannot be reliably detected in zebrafish lysates) and p62 also suggested autophagy induction and enhanced autophagic flux. Co-treatment with spermidine and chloroquine, an autophagy inhibitor, demonstrated that spermidine treatment induces autophagy (higher LC3II levels) and blocking autophagy diminished the improved swimming of the MJD zebrafish following spermidine treatment. Collectively, these findings suggest that spermidine can induce autophagy in the MJD zebrafish, aiding the clearance of mutant ataxin-3 protein species, and providing neuroprotective benefits, aligning with previous findings exploring the utility of spermidine for neurodegenerative diseases [25]. Next, we explored whether spermidine treatment could produce beneficial effects over a longer time course and within a mammalian model. To do this, we explored the effect of long-term spermidine treatment on the CMVMJD135 mouse model of MJD [20].

Western blot analysis of lysates from mice cerebellum collected at the end of the study revealed that spermidine treatment had not affected the amount of human ataxin-3 expressed within the brains of the mice. However, previous studies have also treated mice with the same concentration (3 mM) and treatment route (drinking water) of spermidine and demonstrated delivery to the CNS and removal of soluble Aβ protein (despite no insoluble Aβ plaque removal) [26]. With regards to markers of autophagy induction, it is difficult to conclude without co-treatment with an autophagy blocker in the mice, due to the dynamic nature of the autophagy process. Nevertheless, within our study we found that spermidine treatment significantly increased the amount of phosphorylated ULK1, which has been reported to occur downstream of AMPK in the process of triggering autophagy induction [27]. This finding, together with previous findings of autophagy induction in mice following administration of 3 mM spermidine within drinking water, leads us to propose that the treatment was indeed inducing autophagy as hypothesised. A caveat within our investigation was that we performed our western blot analysis for soluble ataxin-3 levels and autophagy markers on lysates extracted from the cerebellum of the mice. Whilst we counted some ataxin-3 aggregates within the DCN of the cerebellum, far more were counted within the pons and medulla of the mice. This indicates that examination of the medulla oblongata or pons for soluble ataxin-3 abundance would be valuable. Likewise, investigation of autophagy induction may have been valuable within that region in addition or instead of the cerebellum.Administration of spermidine treatment through drinking water did not produce any therapeutic benefit in CMVMJD135 mice. We found no improvement in neurological symptoms or performance in motor behaviour tests including rotarod or balance beam throughout the 13 weeks of treatment administration. Furthermore, whilst the CMVMJD135 mice were found to have more ataxin-3-positive protein aggregates than wild type mice within the pons, medulla oblongata or deep cerebellar nuclei, spermidine treatment had no beneficial effect on the presence of protein aggregates in these regions. This result is interesting, considering that our findings did suggest that the spermidine treatment was producing some changes in line with autophagy induction within the cerebellum of the mice. Our results are in line with the findings of Freitag et al., 2022, who found that spermidine treatment did not produce a reduction in beta-amyloid plaques within their Alzheimer’s disease mouse model [25]. The findings are also in line with previous reports by Duarte-Silva et al. (2014) that induction of autophagy through treatment with lithium did not alleviate motor symptoms in the same mouse model of MJD as that used here [28]. Further, Santana et al. (2020) have reported that whilst treatment with trehalose induced autophagy and decreased motor impairment in a mouse model of MJD, it decreased the size but not the number of ataxin-3 aggregates within lobule IX of the cerebellum [29]. Finally, an additional mouse model of MJD develops motor impairment without the presence of intranuclear ataxin-3 aggregates, suggesting that they might not be pivotal to the pathogenesis of the disease.

In contrast with our findings, previous studies have demonstrated a range of potential therapeutic benefits following spermidine treatment. Xu et al. (2020) reported that administration of spermidine within drinking water to a rodent model of accelerated aging resulted in autophagy induction and improved novel object recognition and open field test behaviours [27]. Wang et al. (2012) found that spermidine treatment of a 7-month-old transgenic TDP-43 mouse model of frontotemporal-dementia produced autophagy induction together with improved rotarod endurance, decreased protein aggregates and decreased neuronal loss [25]. Our study differed from these previous reports in that Xu et al. administered the spermidine orally (0.78 mg/kg/d, rather than in drinking water) and Wang et al. administered the spermidine via intraperitoneal injection (50 mg/kg), three times a week for a month.

In addition to an absence of protective effects, and even poorer neurological scores at some weeks following spermidine treatment of the CMVMJD135 mice, we found that wild-type mice treated with spermidine developed a decreased latency to fall from the accelerating rotarod compared to those administered normal drinking water. We confirmed that these shorter rotarod times were not being triggered by a higher body weight in the spermidine treated (compared to water treated) wild type mice, with instead a trend towards decreased weight seen in the spermidine treated wild type mice. We explored whether spermidine treatment was not only inducing autophagy, but also triggering apoptosis, a related pathway that can result from strong induction of the autophagy pathway. We did not find any signs of increased apoptosis in the immunoblot analysis of the spermidine treated mice cerebellum. However, we did identify signs of increased apoptosis in spermidine-treated zebrafish. We used an acridine orange stain to examine any altered signs of apoptosis produced by 24 h of spermidine treatment of each of the three genotypes, wild type, Ataxin-3 23Q-BFP and Ataxin-3 84Q-BFP. We found increased acridine orange staining in wild-type zebrafish larvae treated with spermidine, compared to vehicle treated wild-type controls, suggesting that spermidine treatment had produced an increase in the number of apoptotic cells present. Whilst there was also elevated acridine orange staining in the spermidine treated Ataxin-3 23Q and Ataxin-3 84Q zebrafish, the level of staining was not significantly increased compared to their respective vehicle treated controls. Additionally, spermidine treatment of wild type zebrafish larvae resulted in increased cleaved PARP1 and cleaved caspase-3, both signs of increased apoptosis, in protein lysates. Whilst we were not able to identify signs of increased apoptosis in western blots performed on the spermidine treated wild type mice, this may be due to the semi-quantitative nature of western blot analysis and does warrant further investigation via other methods.

Whilst this is the first report of spermidine treatment producing increased apoptosis within a neurological setting, references to a link between spermidine treatment and apoptosis can be found within the literature [30, 31]. Studies have previously proposed that spermidine may have benefit as a cancer treatment due to its ability to induce apoptosis (indicated by increased Annexin V staining), together with autophagy induction, in HeLa cells (a cervical cancer cell line) [30] and human breast cancer cells [31]. In contrast with those findings, Kim et al. (2021) previously reported that spermidine treatment reduced peroxide induced apoptosis within retinal pigment epithelial cells [32] and Xu et al. (2020) reported decreased Bcl-2, Bax and caspase-3 following spermidine treatment in rodent model of aging-induced dementia [27]. Certainly, closer investigation into whether spermidine treatment induces apoptotic activity in neurons in vitro and following in vivo treatment, would be valuable.

Together, these findings suggest that whilst spermidine treatment is capable of inducing autophagic activity, the therapeutic benefit of this treatment is not sufficient to significantly attenuate the development of MJD. Treatment did not prevent development of protein aggregates in the CMVMJD135 mice or prevent movement dysfunction in the animals. Furthermore, we observed some negative signs following spermidine treatment of non-transgenic animals, including decreased rotarod endurance in mice and increased signs of apoptosis in zebrafish larvae. These findings suggest that a cautious approach should be taken regarding the use of spermidine, a supplement previously proposed to be capable of inducing longevity. We recommend that additional studies into the safety of spermidine administration, particularly in healthy subjects, are needed before further therapeutic application.

### Electronic supplementary material

Below is the link to the electronic supplementary material.

Supplementary Material 1

## Data Availability

The dataset supporting the conclusions of this article is included within the article (and its additional files).
