# Supplementary material for: Spermidine treatment: induction of autophagy but also apoptosis?
Source: Mol Brain. 2024 Mar 5;17:15. doi: 10.1186/s13041-024-01085-7 (PMC10916058; doi:10.1186/s13041-024-01085-7)
Supplement: Supplementary file 1 — Supplementary Material 1 [file 13041_2024_1085_MOESM1_ESM.docx]

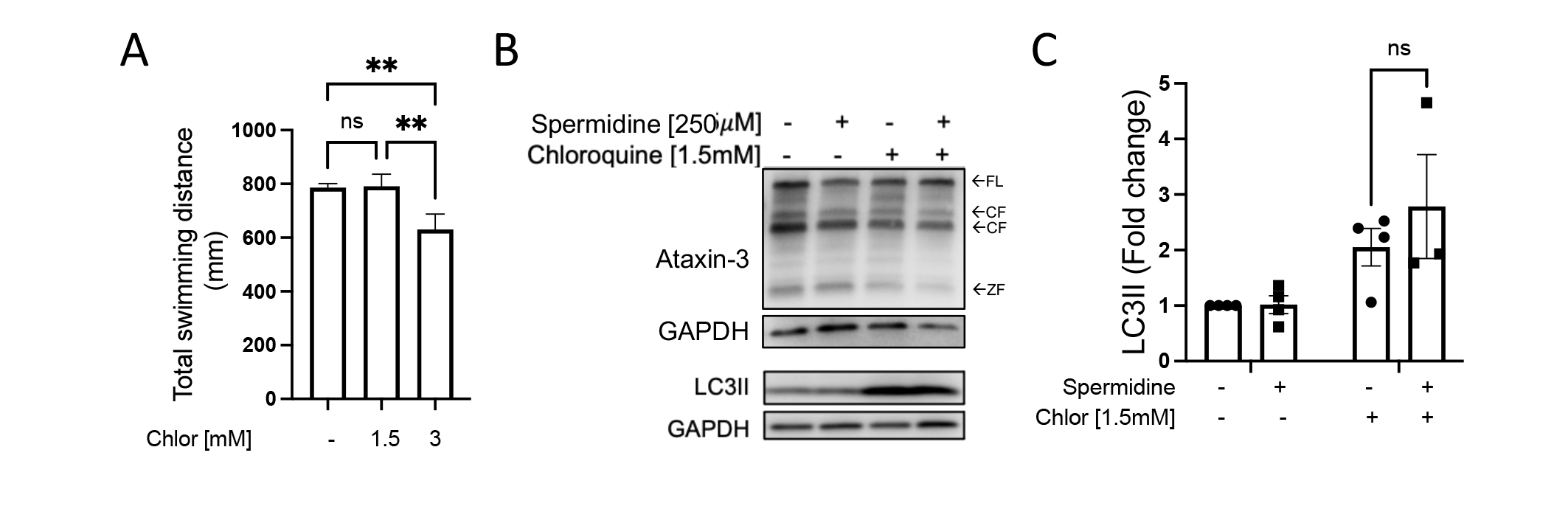


**Supplementary Figure 1.** Lower concentration of chloroquine does not inhibit spermidine mediated autophagic flux. A) Non-transgenic zebrafish treated with 1.5 mM and 3 mM chloroquine showed impaired locomotion with 3 mM chloroquine compared to the lower concentration of chloroquine and the vehicle control (p < 0.0097). B) Representative western blot of MJD zebrafish treated with either spermidine or chloroquine (1.5 mM) revealed that spermidine and chloroquine co-treatment failed to increase LC3II levels compared to chloroquine treatment alone and this is confirmed in the C) quantification of LC3II levels.


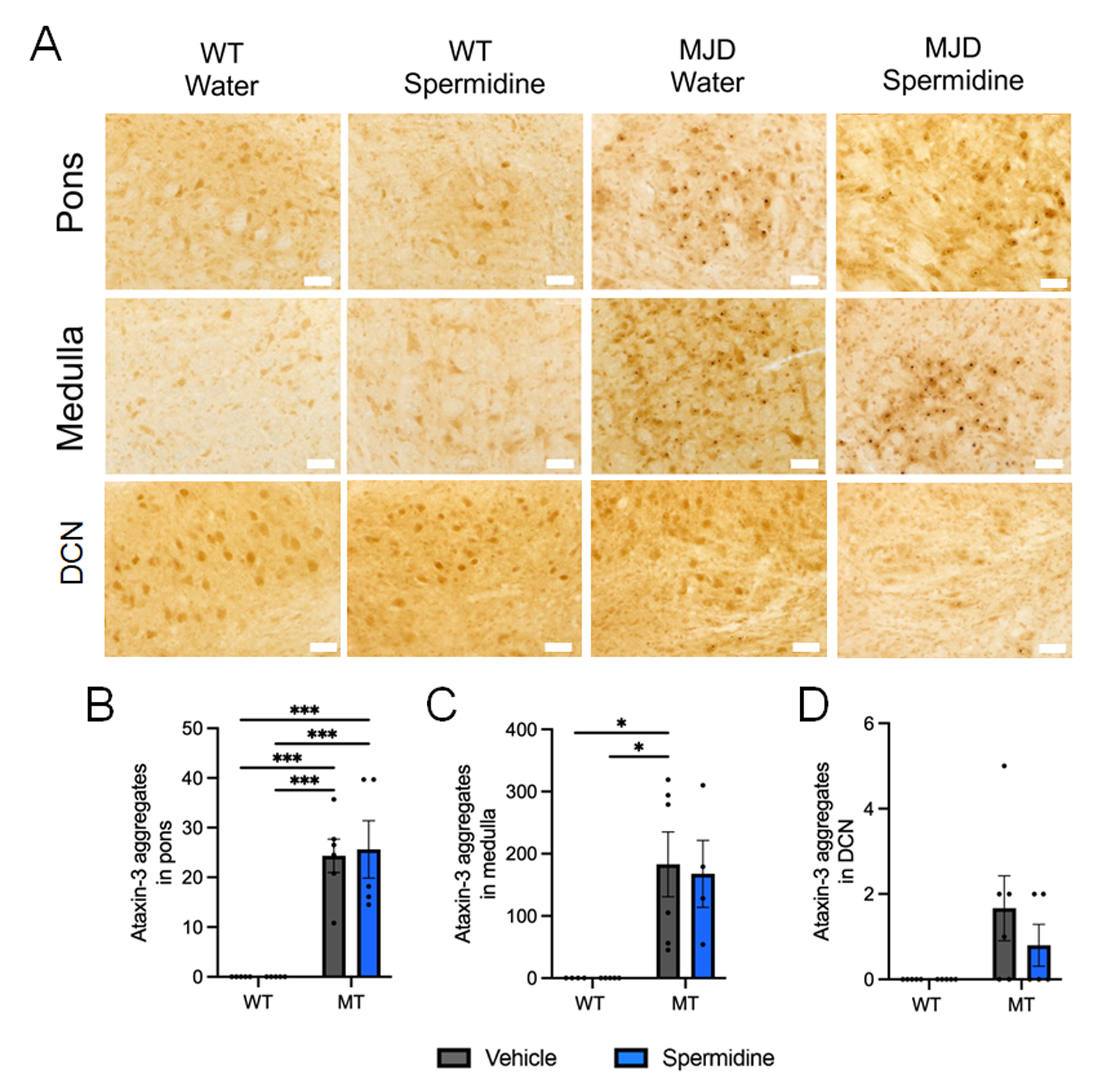


**Supplementary Figure 2. Spermidine treatment did not affect presence of protein aggregates within the pons, medulla oblongata or deep cerebellar nuclei (DCN) of 25-week-old CMVMJD135 mice.** A) Immunohistochemical staining for ataxin-3 in sections from the pons, medulla oblongata and deep cerebellar nuclei region of wild type (WT) and CMVMJD135 (MJD) mice revealed that the MJD mice harboured ataxin-3-positive protein aggregates, particularly within the pons and medulla oblongata. Scale bars indicate 50 µm. B) MJD mice exhibited more ataxin-3 aggregates than WT mice within the pons (p < 0.0001) and spermidine treatment had no effect on the number of ataxin-3 aggregates present. C) MJD mice exhibited more ataxin-3 aggregates within the medulla oblongata than WT mice (p=0.0005) and spermidine treatment had no effect on the number of ataxin-3 aggregates present. D) More aggregates were found within the DCN region in MJD mice than WT mice (p = 0.0482), but no benefit of spermidine treatment was found. A two-way ANOVA was utilised for statistical analysis followed by Tukey post hoc. The p-values marked on the graphs represent the Tukey test results. Data represents mean ± SEM.
